# Supplementary figures and images for: Mutations in chikungunya virus nsP4 decrease viral fitness and sensitivity to the broad-spectrum antiviral 4′-Fluorouridine
Source: PLoS Pathog. 2025 Jan 13;21(1):e1012859. doi: 10.1371/journal.ppat.1012859 (PMC11759387; doi:10.1371/journal.ppat.1012859)

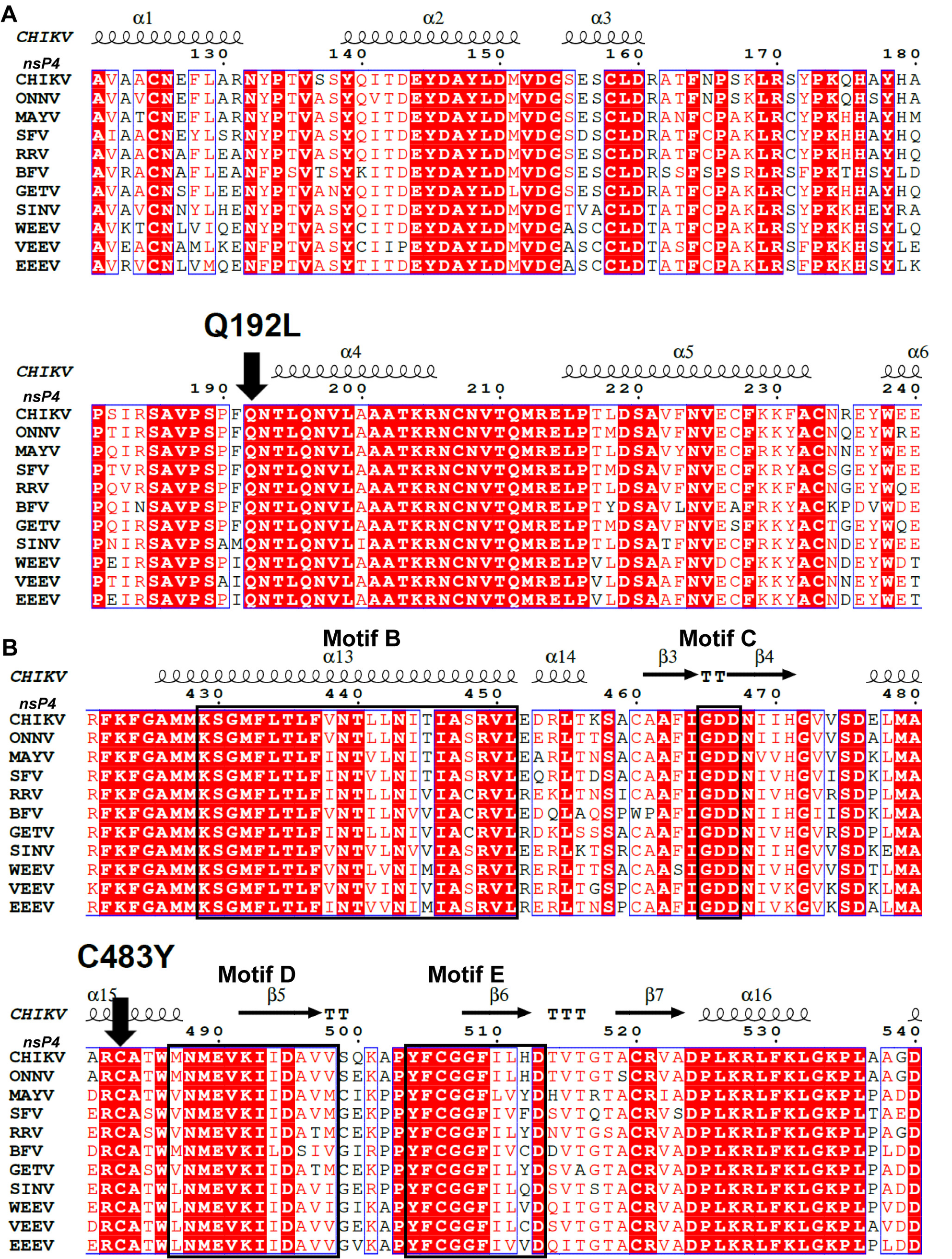

Supplement: S1 Fig — Alignment of alphavirus nsP4 sequences around residues (A) Q192 or (B) C483. Q192 and C483 are indicated by arrows, and the motifs in the nsP4 palm domain are marked by black boxes at the top of the sequences. The blue boxes show the conserved regions. TT stands for tight turn. Swiss-Prot accession numbers: CHIKV: A4L7I2, ONNV: P13886, MAYV: Q8QZ73, SFV: P08411, RRV: P13887, BFV: P87515, GETV: Q5Y389, SINV: P03317, WEEV: P13896, VEEV: P36328, EEEV: Q306W6. (TIF) [file ppat.1012859.s001.tif]

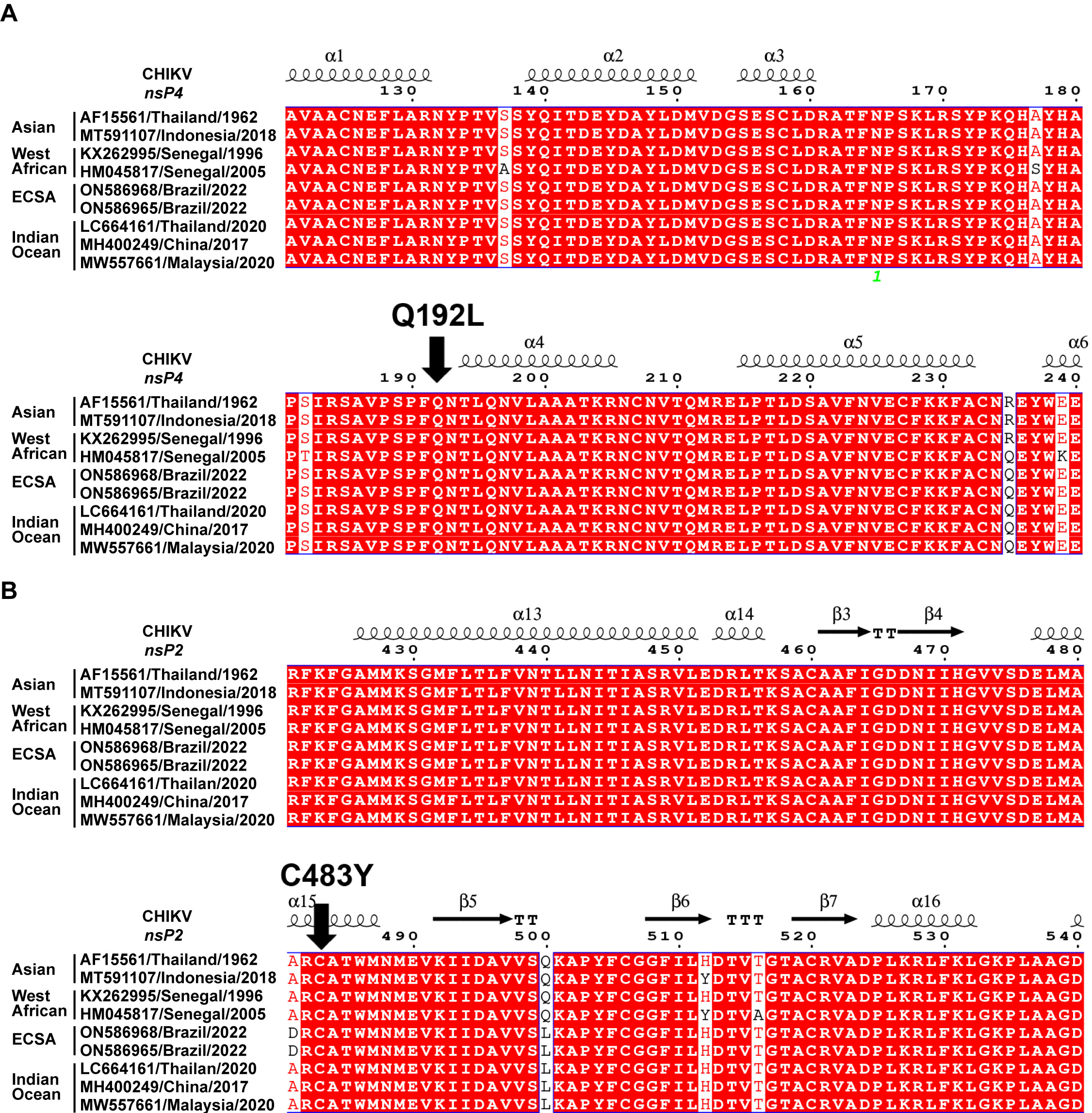

Supplement: S2 Fig — Alignment of nsP4 sequences across CHIKV strains around residues (A) Q192 or (B) C483. Q192 and C483 are indicated by arrows. The blue boxes indicate conserved regions. TT stands for tight turn. GenBank numbers are indicated; the GenBank number for strain AF15561 is EF452493. (TIF) [file ppat.1012859.s002.tif]

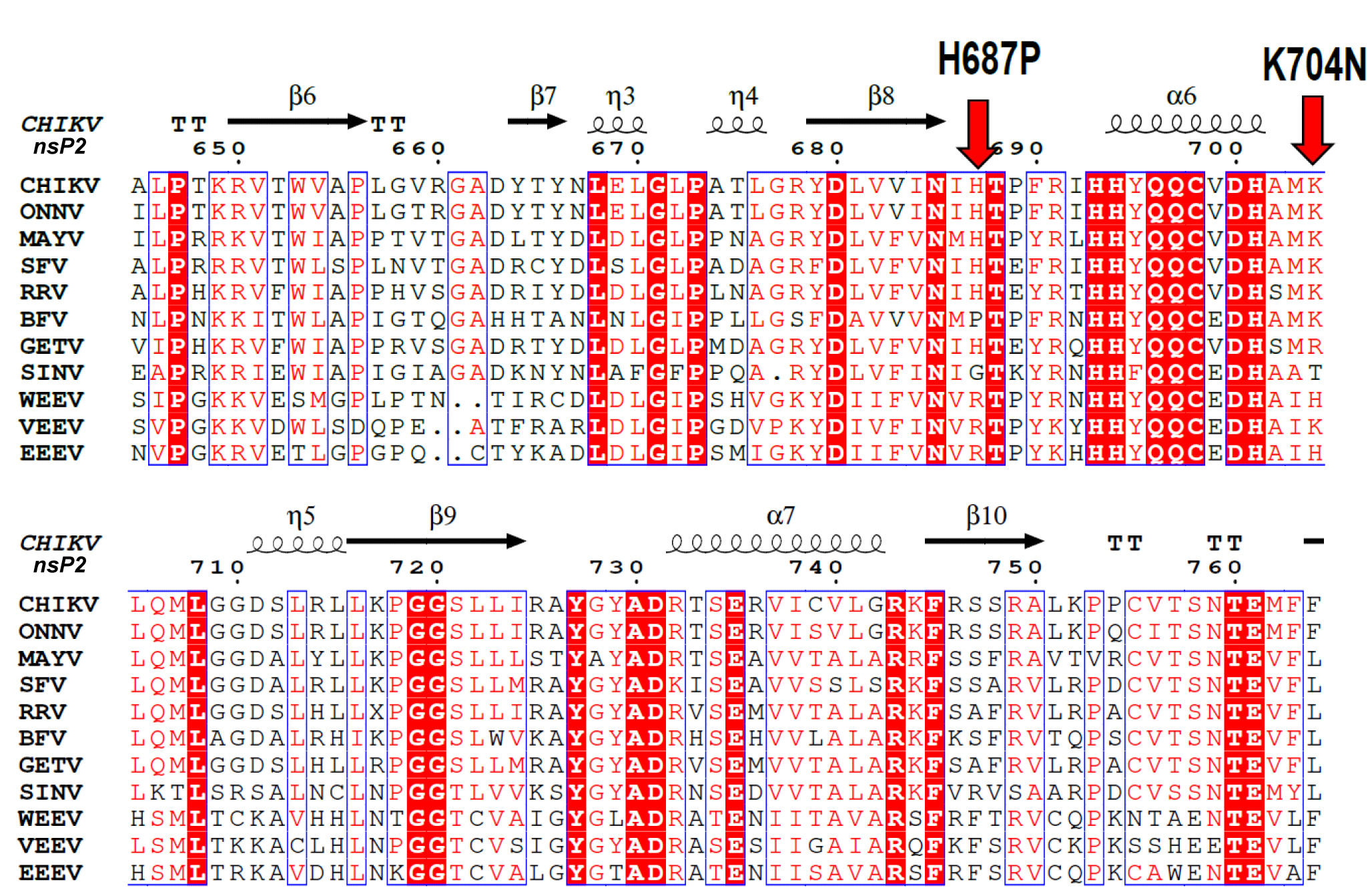

Supplement: S3 Fig — H687 and K704 are indicated by arrows. The blue boxes indicate conserved regions. TT stands for tight turn. The Swiss-Prot accession numbers are the same as in S1 Fig. (TIF) [file ppat.1012859.s003.tif]

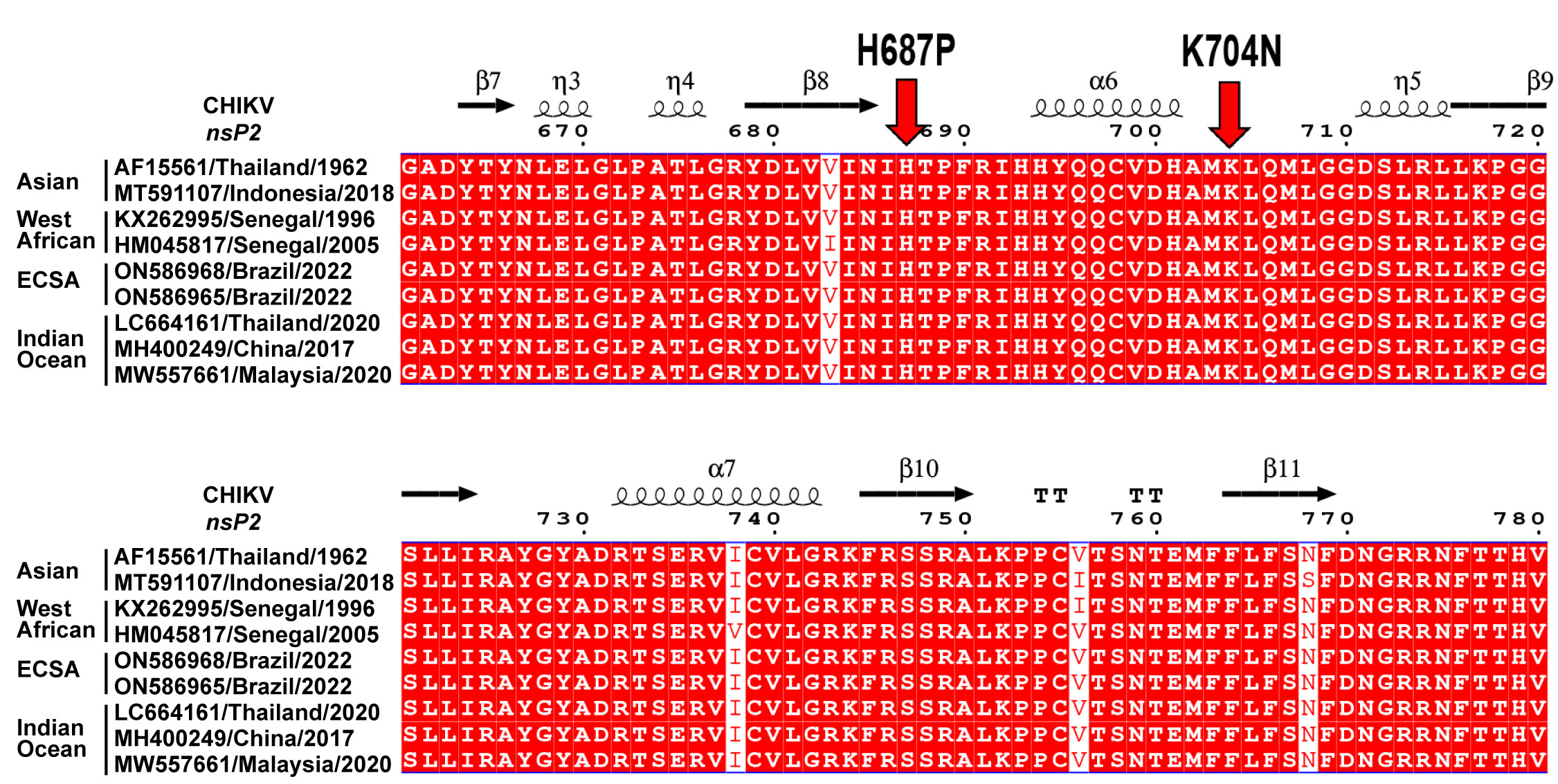

Supplement: S4 Fig — H687 and K704 are indicated by arrows. The blue boxes indicate conserved regions. TT stands for tight turn. GenBank numbers are indicated; the GenBank number for strain AF15561 is EF452493. (TIF) [file ppat.1012859.s004.tif]

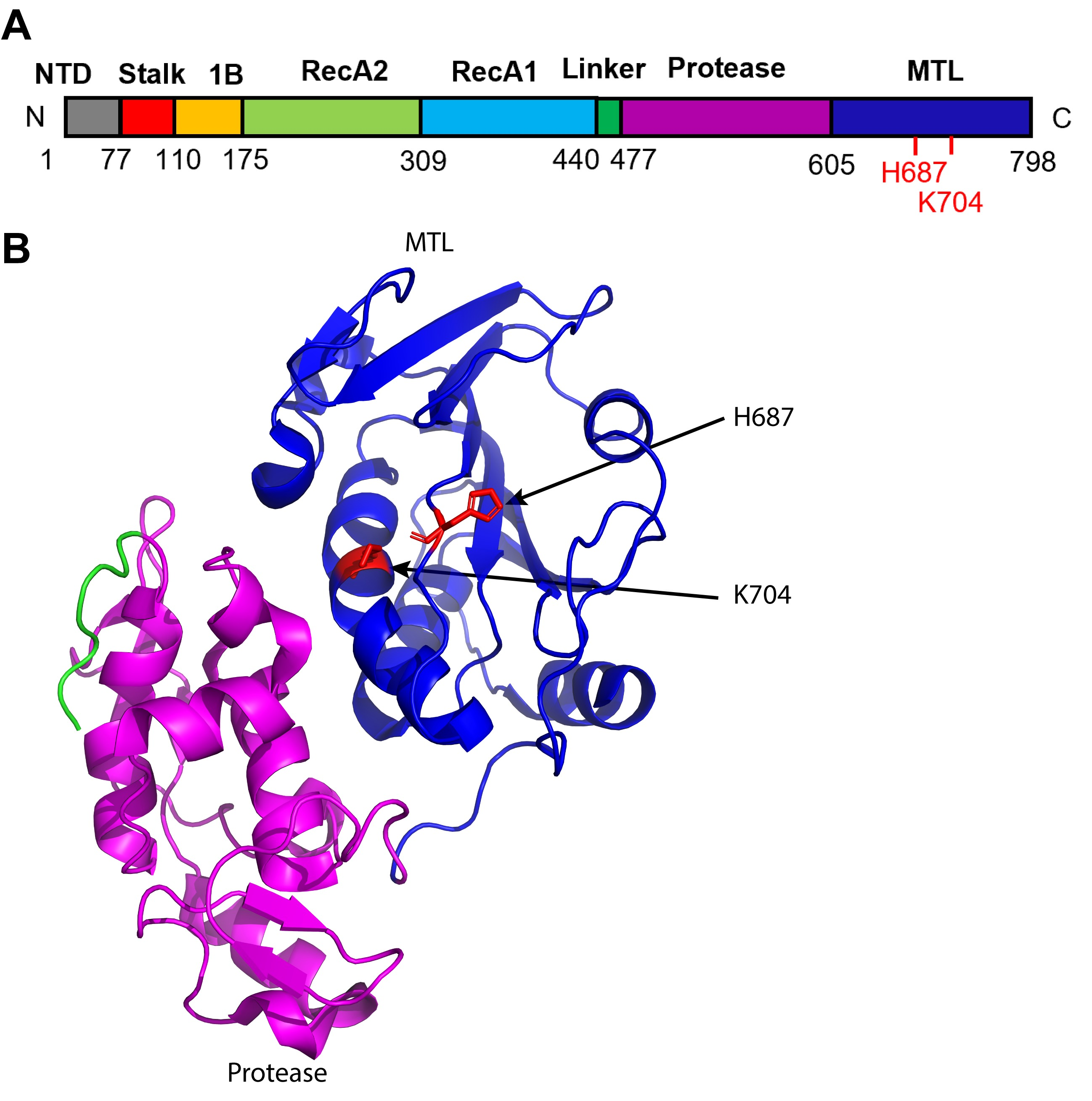

Supplement: S5 Fig — (A) Linear diagram of CHIKV nsP2 sequence, indicating the domains and the positions of the H687 and K704 residues. (B) Structure of the nsP2 protease and MTL domains, indicating the positions of H687 and K704. PDB:4ZTB [23]. (TIF) [file ppat.1012859.s005.tif]

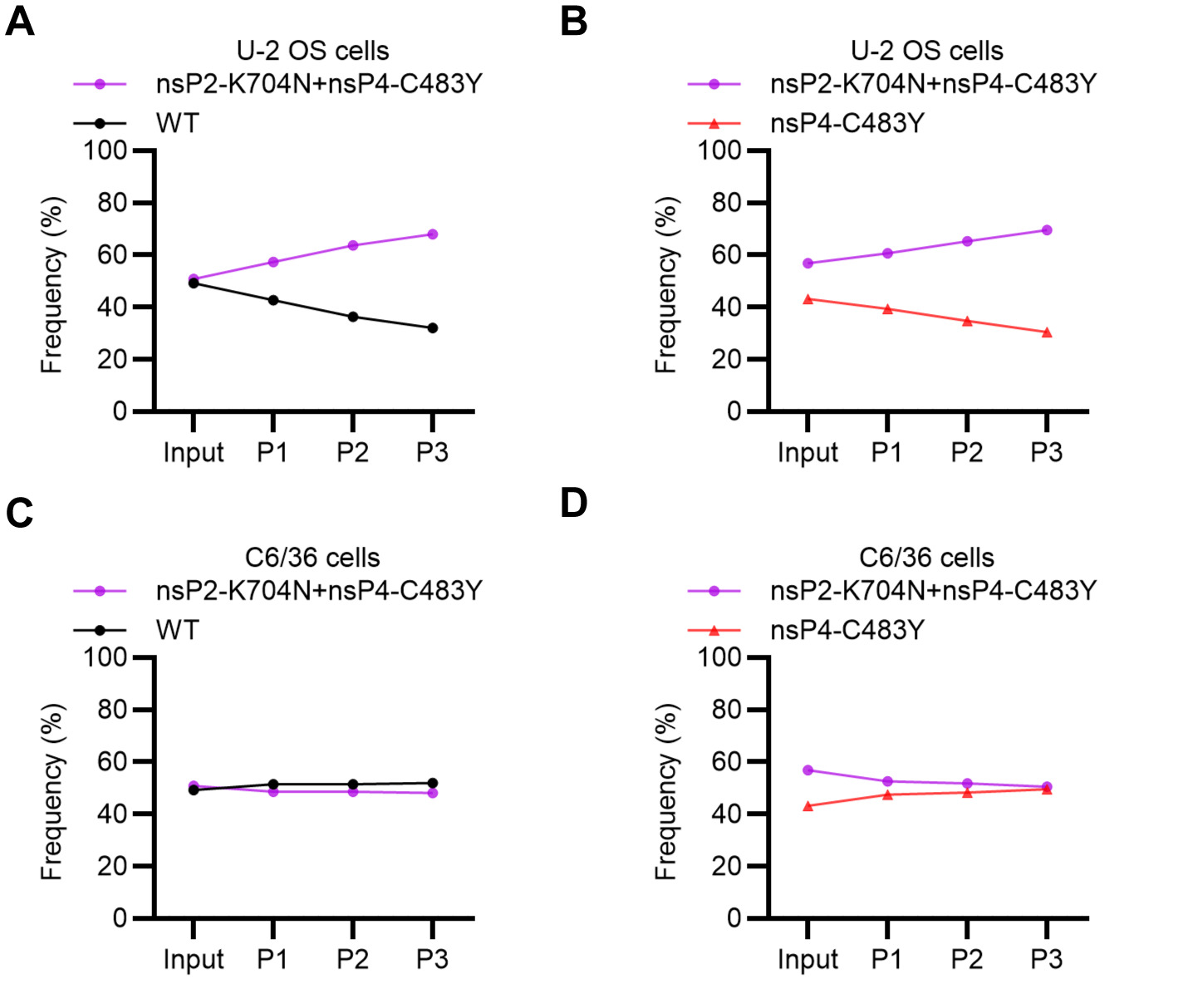

Supplement: S6 Fig — (A and B) Competition assays comparing relative viral fitness in U-2 OS cells. Cells were inoculated and viruses serially passaged and analyzed as in Fig 4A. (C and D) Competition assays comparing relative viral fitness in C6/36 cells. Cells were inoculated and viruses serially passaged and analyzed as in Fig 4D. Panels A and C report the frequency of nsP4-C483Y to represent the nsP2-K704N+nsP4-C483Y mutant. Panels B and D report the frequency of nsP2-K704N to distinguish the two competitors. (TIF) [file ppat.1012859.s006.tif]
