# Supplementary material for: Mutations in chikungunya virus nsP4 decrease viral fitness and sensitivity to the broad-spectrum antiviral 4′-Fluorouridine
Source: PLoS Pathog. 2025 Jan 13;21(1):e1012859. doi: 10.1371/journal.ppat.1012859 (PMC11759387; doi:10.1371/journal.ppat.1012859)
Supplement: S1 Table — (DOCX) [file ppat.1012859.s007.docx]

**S1 Table. Summary of mutations with** **frequency >20% in CHIKV lineages**

^a^ The number of sequencing reads at the indicated locus.

^b^ Shown are the mutations present in the indicated independent lineages after six passages in the presence of 4′-FlU. The protein and amino acid change are listed. Mutations also present in plaque-purified viruses derived from the indicated lineage are highlighted in bold (see S2 Table).

| **Sample** | **Frequency (%)** | **Change type** | **Sequencing**  **Depth^a^** | **AA change^b^** | **Nucleotide change** |
| --- | --- | --- | --- | --- | --- |
| 4′-FlU  lineage #1 | 26.76 | nonsynonymous | 4868 | nsp1: A493V | nsp1: C1478T |
|  | 27.75 | synonymous | 4118 | nsp2: G279G | nsp2: T837C |
|  | **48.71** | **nonsynonymous** | **3182** | **nsp2: K704N** | **nsp2: A2112C** |
|  | **89.92** | **nonsynonymous** | **5341** | **nsp4: C483Y** | **nsp4: G1466A** |
| 4′-FlU  lineage #2 | 28 | nonsynonymous | 3798 | nsp2: H687P | nsp2: A2060C |
|  | **36.47** | **nonsynonymous** | **4471** | **nsp4: Q192L** | **nsp4: A593T** |
|  | **51.8** | **nonsynonymous** | **5037** | **nsp4: C489Y** | **nsp4: G1466A** |
| 4′-FlU  lineage #3 | **62.92** | **nonsynonymous** | **3653** | **nsp2: K704N** | **nsp2: A2112C** |
|  | **91.93** | **nonsynonymous** | **5645** | **nsp4: C483Y** | **nsp4: G1466A** |
| 4′-FlU lineage #4 | 19.95 | nonsynonymous | 4302 | nsp2: H687P | nsp2: A2060C |
|  | 23.79 | nonsynonymous | 3806 | nsp2: K704N | nsp2: A2112C |
|  | **25.1** | **nonsynonymous** | **4728** | **nsp4: Q192L** | **nsp4: A593T** |
|  | **68.4** | **nonsynonymous** | **5205** | **nsp4: C483Y** | **nsp4: G1466A** |
| 4′-FlU  lineage #5 | 29.29 | synonymous | 4281 | nsp4: T376T | nsp4: G1128T |
|  | 30.66 | nonsynonymous | 5967 | nsp2: A57S | nsp2: G169T |
|  | 31.32 | nonsynonymous | 4995 | nsp2: K507Q | nsp2: A1519C |
|  | **45.7** | **nonsynonymous** | **3737** | **nsp2: K704N** | **nsp2: A2112C** |
|  | **92.92** | **nonsynonymous** | **5043** | **nsp4: C483Y** | **nsp4: G1466A** |
| 4′-FlU  lineage #6 | **43.05** | **nonsynonymous** | **3425** | **nsp2: K704N** | **nsp2: A2112C** |
|  | **83.22** | **nonsynonymous** | **5804** | **nsp4: C483Y** | **nsp4: G1466A** |
