# Supplementary material for: Mutations in chikungunya virus nsP4 decrease viral fitness and sensitivity to the broad-spectrum antiviral 4′-Fluorouridine
Source: PLoS Pathog. 2025 Jan 13;21(1):e1012859. doi: 10.1371/journal.ppat.1012859 (PMC11759387; doi:10.1371/journal.ppat.1012859)
Supplement: S2 Table — (DOCX) [file ppat.1012859.s008.docx]

**S2 Table. Summary of mutations in plaque-purified 4′-FlU-selected lineages.**

| **Sample** | **Frequency (%)** | **AA Change ^a^** | **Depth** | **Nucleotide change** | **Observed in lineage?** |
| --- | --- | --- | --- | --- | --- |
| 4′-FlU-PP 1 | **99.93** | **nsp4: C483Y** | **11844** | **nsp4: G1448A** | **Yes (89.92%)** |
|  | 99.91 | nsp3: E154K | 8745 | nsp3: G460A | No |
|  | 99.91 | E1: S323I | 8667 | E1: G968T | No |
|  | **99.86** | **nsp2: K704N** | **5634** | **nsp2: A2112C** | **Yes (48.71%)** |
| 4′-FlU-PP 2 | **99.92** | **nsp2: H687P** | **5901** | **nsp2: A2060C** | **Yes (28%)** |
|  | **99.85** | **nsp4: Q192L** | **8109** | **nsp4: A575T** | **Yes (36.47%)** |
| 4′-FlU-PP 3 | **99.97** | **nsp4: C483Y** | **11868** | **nsp4: G1448A** | **Yes (91.93%)** |
|  | **99.93** | **nsp2: K704N** | **5920** | **nsp2: A2112C** | **Yes (62.92%)** |
| 4′-FlU-PP 4 | **99.85** | **nsp2: H687P** | **5929** | **nsp2: A2060C** | **Yes (19.95%)** |
|  | 99.83 | nsp4: K81T | 10683 | nsp4: A242C | No |
|  | **99.65** | **nsp4: Q192L** | **8113** | **nsp4: A575T** | **Yes (25.1%)** |
| 4′-FlU-PP 5 | **99.91** | **nsp4: C483Y** | **12520** | **nsp4: G1448A** | **Yes (92.92%)** |
|  | 99.9 | nsp3: Q301R | 10123 | nsp3: A902G | No |
| 4′-FlU-PP 6 | 99.95 | nsp3: N75K | 9720 | nsp3: T225A | No |
|  | **99.9** | **nsp4: C483Y** | **13075** | **nsp4: G1448A** | **Yes (83.22%)** |
| DMSO-PP 1 | 99.94 | nsp2: G554E | 10552 | nsp2: G1661A | No |
| DMSO-PP 2 | 91.97 | nsp3: K188N | 7866 | nsp3: A564C | No |
|  | 91.72 | nsp2: D351G | 7116 | nsp2: A1052G | No |
| DMSO-PP 3 | 99.97 | nsp3: K188E | 7706 | nsp3: A562G | No |

^a^ Mutations detected in plaque-purified viruses derived from 4′-FlU and DMSO lineages after 6 passages in the presence of 4′-FlU. Mutations also observed in lineages (see S1 Table) are highlighted in bold.
