# Supplementary material for: Mutations in chikungunya virus nsP4 decrease viral fitness and sensitivity to the broad-spectrum antiviral 4′-Fluorouridine
Source: PLoS Pathog. 2025 Jan 13;21(1):e1012859. doi: 10.1371/journal.ppat.1012859 (PMC11759387; doi:10.1371/journal.ppat.1012859)
Supplement: S3 Table — (DOCX) [file ppat.1012859.s009.docx]

**S3 Table. Primers used to generate mutations in CHIKV infectious cDNA clones**

| **Mutant** | **Primer name** | **Sequence** |
| --- | --- | --- |
| nsP2-H687P | Age1-F1 | CTACCACCAACATTATACCGGTCAACAGGAGACTACC |
|  | nsP2-H687P-R1 | GTGTATGCGAAAAGGTGTGGGGATGTTTATGACCACTAG |
|  | nsP2-H687P-F2 | CTAGTGGTCATAAACATCCCCACACCTTTTCGCATACAC |
|  | Swa1-R2 | GCTAACGGTTTGCCCAATTTAAATAACCTTTTTAGCGGG |
| nsP2-K704N | Age1-F1 | CTACCACCAACATTATACCGGTCAACAGGAGACTACC |
|  | nsP2-K704N-R1 | CCCCCTAGCATTTGCAGGTTCATTGCGTGATCTACG |
|  | nsP2-K704N-F2 | CGTAGATCACGCAATGAACCTGCAAATGCTAGGGGG |
|  | Swa1-R2 | GCTAACGGTTTGCCCAATTTAAATAACCTTTTTAGCGGG |
| nsP4-Q192L | Age1-F1 | CTACCACCAACATTATACCGGTCAACAGGAGACTACC |
|  | nsP4-Q192L-R1 | CATTCTGTAGTGTGTTCAGGAATGGGGAAGGTACAGC |
|  | nsP4-Q192L-F2 | GCTGTACCTTCCCCATTCCTGAACACACTACAGAATG |
|  | Swa1-R2 | GCTAACGGTTTGCCCAATTTAAATAACCTTTTTAGCGGG |
| nsP4-C483Y | Age1-F1 | CTACCACCAACATTATACCGGTCAACAGGAGACTACC |
|  | nsP4-C483Y-R1 | CATGTTCATCCAAGTAGCGTATCTGGCTGCCATCAATTC |
|  | nsP4-C483Y-F2 | GAATTGATGGCAGCCAGATACGCTACTTGGATGAACATG |
|  | Swa1-R2 | GCTAACGGTTTGCCCAATTTAAATAACCTTTTTAGCGGG |
