# Supplementary material for: Mutations in chikungunya virus nsP4 decrease viral fitness and sensitivity to the broad-spectrum antiviral 4′-Fluorouridine
Source: PLoS Pathog. 2025 Jan 13;21(1):e1012859. doi: 10.1371/journal.ppat.1012859 (PMC11759387; doi:10.1371/journal.ppat.1012859)
Supplement: Attachment — Submitted filename: PLoS Path_2749 Resistance_Response_final.pdf [file ppat.1012859.s017.pdf]

RESPONSE TO REVIEWERS
Resubmission of PLoS Pathogens D-24-01851

We appreciate the detailed comments and thoughtful suggestions of the three reviewers of our manuscript. We are pleased that the reviewers found that our paper was “well performed…..clearly written” and “of interest to the readers of PLoS Pathogens”. The reviewers made a number of useful and constructive suggestions, which we have addressed in full as detailed below. Their points are given first, followed by our responses in blue.

REVIEWER 1

In this manuscript, Yin et al. assess the potential of the ribonucleoside analog 4’Fluorouridine (4’-FIU) as effective antiviral against CHIKV by characterizing the in vitro resistance profile of CHIKV to the compound and testing whether the resistance mutations alter viral fitness in vitro and in vivo. CHIKV mutants less sensitive to 4’-FIU were selected during multiple rounds of passaging under 4’-FIU pressure in U-2 OS cells. Different sets of mutations were detected by Illumina whole genome sequencing, from which two pairs of mutations in nsP2 and nsP4 were most prevalent. Two mutations in nsP4 were responsible for the lower sensitivity to 4’-FIU, i.e., nsP4-C483Y and nsP4-Q192L. They then determined whether these mutations altered viral fitness in vitro and in vivo. NsP4-C483Y virus remained as fit as WT CHIKV, whereas the nsP4-Q192L mutation decreased viral fitness. Additionally, the authors performed RdRp fidelity analyses, because nsP4-C483Y was previously shown to alter polymerase fidelity. However, no significant differences between WT, nsP4-C483Y, and nsP4-Q192L were noted. Taken together, these data confirm previously published data on nsP4-C483Y regarding viral fitness and provide new insights on the novel mutation nsP4-Q192L. However, the most important conclusion in the manuscript is not supported by statistical analysis. Furthermore, the mutations nsP4-C483Y and nsP4-Q192L were characterized as stand-alone entities, whereas nsP4-Q192L was detected in conjunction with nsP2-H687P, which could potentially be a compensatory mutation. Hence, the manuscript should be reinforced with novel experimental data as outlined below.

Major Comments

1. The conclusion that nsP4 mutations nsP4-C483Y and nsP4-Q192L are primarily responsible for the reduction in sensitivity to 4’-FIU is based on the dose-response curves in Fig.2A+B, but no statistical analysis was performed whilst the variation is visibly high. The authors should perform statistics on the EC90 analysis and show the individual data points in Fig.2B. If the differences are not significant due to the high variation, the authors should amend the main text to phrase the results in a less strong fashion. Has the same set of nsP2 and nsP4 mutants been included in the trans-replication assay (Fig. 3C,D) and the dose-response curve in human dermal fibroblasts (Fig. 6A), although not shown in the manuscript? Please, include the whole set of mutants (from Fig.2) in these two assays to support the main conclusions from the manuscript by orthogonal methodology.

- Thank you for these comments. We do see some variability in these virus production assays, and have therefore repeated the dose response assay in Fig. 2A two more times. We then performed statistical analysis on all of the data and also included the individual data points in Fig. 2B. Together the results indicated that the nsP4 mutations nsP4-C483Y and nsP4-Q192L are primarily responsible for the reduction in sensitivity to 4′-FIU. Please see the updated Fig. 2A and 2B.
- After confirming the primary role of nsP4-C483Y and nsP4-Q192L in reduced sensitivity to 4′-FIU, we focused on these two nsP4 mutations in the rest of the experiments. Since the nsP2 mutations did not confer a significant reduction in 4′-FIU sensitivity either alone or together with the nsP4 mutations, the nsP2 mutants were not further evaluated in the trans-replicase assay or the dose-response assay in human dermal fibroblasts.

2. Viral fitness of nsP4-C483Y and nsP4-Q192L was assessed in growth curves and competition assays in vitro and in vivo. What was the reason why the nsP4 mutants were characterised as stand-alone entities and not in context of the other mutations detected by whole genome sequencing? NsP4-Q192L, which results in a reduction in viral fitness, has only been detected in conjunction with nsP2-H687P. The effect of nsP2-H687P on the viral

fitness of nsP4-Q192L should be assessed (at least in vitro) to confirm or negate that nsP2-H687P is a compensatory mutation restoring viral fitness of nsP4-Q192L to WT levels.

- We performed new competition experiments in U-2 OS cells and C6/36 mosquito cells to test if the nsP2-H687P mutation helps to rescue the fitness of nsP4-Q192L (Fig.4A, 4B, 4E, 4F). The nsP4-Q192L mutant and the nsP2-H687P-nsP4-Q192L double mutant were similarly outcompeted by CHIKV 181/25 WT in both cell types. We also directly compared the fitness of the nsP4-Q192L mutant with the nsP2-H687P-nsP4-Q192L double mutant (Fig. 4C, 4G). We found that the frequency of nsP2-H687P-nsP4-Q192L was actually somewhat decreased vs. nsP4-Q192L mutant. Together these data indicate that the nsP2-H687P mutation did not help to rescue the fitness of nsP4-Q192L. We think these new data add to the manuscript, and thank the reviewer for the suggestion.

3. Although the manuscript is written comprehensively, it is not supported by intuitive and to-the-point figures. The figures contain too much white space, whereas titles of graphs and legends often contain redundant information (for example, adding CHIKV to all figure legends is unnecessary when it is the only virus studied). The authors should streamline the figures and resolve any inconsistencies, see detailed remarks in the Minor Comments section.
- We appreciate the reviewer's comments. We have endeavored to reduce white space in the figures, especially in Fig. 2. We removed the CHIKV label in most figures, but we maintained it in Figure 3 to distinguish virus infection vs. the trans-replicase system, which is based on plasmid transfection. We added an explanation of this in the legend for Figure 3.

4. It is unclear how the trans-replication assay was performed. Figure 3B clearly shows that two plasmids (nsPs + template) were transfected, whereas the Material and Methods states that nsP4 was transfected on a third separate plasmid.

- The trans-replicase assay was performed by transfection of three plasmids. We apologize for the confusion, and clarify this in the legend and the amended Fig. 3B which shows the three plasmids used.

5. Consider placing the section on RdRp fidelity after the section on nsP4 activity (switching Figures 4 and 5). This would divide the manuscript in 'effect on RdRp function' and 'viral fitness assessment', enhancing its clarity for the reader.

- Thank you for the thoughtful suggestion regarding the order of the sections. We carefully considered this arrangement; however, we believe that keeping the current sequence better maintains the logical flow of the manuscript.

6. Is there any evidence that 4'-FIU selects for the here-described CHIKV mutations in vivo during 4'-FIU treatment? Has WGS been performed on the 4'-FIU-treated mice inoculated with WT CHIKV or is this data available from the previously published study initially describing the effectivity of 4'-FIU as CHIKV antiviral (doi: 10.1128/mbio.00420-24)?

- WGS was not performed on the 4′-FIU-treated mice in our previous mBio study. Our in vitro results indicate that the mutations reduce sensitivity to 4′-FIU, but that replication of the mutants is still inhibited at higher concentrations of 4′-FIU. In vitro selection for resistance was done by repeated passages at concentrations below the EC_{90}. Together the data suggest that the doses of 4′-FIU used in our prior studies would remain effective against CHIKV in mice and not select for resistance in the time frame of these experiments.

Minor Comments

1. Title: capitalize the P in nsP4.
- Done.

2. Line 67: assemble 'as' progeny virions.
- Done.

3. Line 92: add that ribavirin is a nucleoside analogue.
- Done.

4. Line 94: please rephrase ‘which has been shown to have effects on RdRp fidelity’, see later comment about lines 227-228.
- We now say “has been characterized for effects on nsP4 fidelity”, leaving this issue open for more detailed discussion later. See also comments on lines 227-228 below.

5. Line 103: what is meant by ‘the’ physiological nucleotide, is there a specific one it mimics?
- We have expanded this to “It mimics the physiological nucleotide uridine and inhibits RNA synthesis by triggering chain termination”.

6. Line 116: add a nuanced reasoning to this conclusion, because the authors do show that CHIKV develops resistance to 4’-FIU, so why is it still a good candidate?
- The CHIKV variants are not completely resistant to 4′-FIU but exhibit only reduced sensitivity to it. This conclusion is supported by the absence of any detectable fitness advantage, as well as no observed increase in disease severity or viral tissue burden.

7. Line 121: add that U-2 OS is a human osteosarcoma cell line.
- Done.

8. Line 129: add that the MOI of the initial primary stock was 0.5 as well.
- Done

9. Line 130: add that just one virus clone was plaque purified and sequenced per lineage.
- Done.

10. Line 132: add that WGS consisted of Illumina deep sequencing to distinguish it from nanopore sequencing.
- Done.

11. Line 140: which threshold was used to define ‘high’?
- The highest mutation in DMSO lineages is an E2 G279E mutation with frequency of ~10%. We have clarified the threshold in the text.

12. Lines 133-159: Consider rewriting this section in a more concise manner.
- We have condensed this section as much as we could while maintaining clarity.

13. Fig 1: Panel A: add that one plaque was purified and sequenced. Panel B: match the connecting line of the DMSO lineages to the symbol (do not use white). Add space between lineage and number. ‘MOI’ is not aligned. Panel C: typo, it says nsP4-Q912L instead of 192. Panels E, G, I: simplify by choosing one colour and show a gradient for the passages. Figure legend: add (red) and (blue) to section describing panel (C). Add space between lineage and number (lines 801 and 803).
- We appreciate the reviewer’s suggestions. For panel A, we have added an explanation in the main text and figure legend. We have changed or corrected the figure as suggested in panel B, C, E and G. We have updated the figure legends.

14. Line 163: ‘resulted in’ instead of ‘produced’.
- Done.

15. Line 166: ‘and’ instead of ‘or’.
- Done.

16. Line 168: as mentioned as Major Comment, rephrase the conclusions with more nuance because no statistics has been performed.

- Additional experiments have now been included and statistical analysis on all of the data performed, see comments to major point 1.

17. Fig 2: Panel B: add individual data points and statistics. Panel C, D: The Y axis title does not state FFU/mL, has another method been performed that has not been described in the Material and Methods? Change the X axis title to Hours Post Inoculation (be consistent in all figures and main text). Panel E: The N and C termini are not aligned. Figure legend: add (nsP2h) in between brackets after 'nsP2 helicase domain'.

- For Panel B, we have added individual data points and statistics. Please also see the response in the main comments. We have updated figure 2 and the legend as suggested for panels C, D and E.

18. Line 194: rephrase, a mutant does not have the ability to decrease something, the mutant results in a decreased sensitivity
- Done.

19. Line 194: change 'precursor' to 'polyprotein' as the polyprotein can already be active prior to nsP2 cleavage.
- Done.

20. Lines 196-199: extend the explanation on what Fluc and Gluc activities represent within the context of the trans-replication assay. Gluc activity is not a sole read-out of 'transcription' because genome replication is required prior to SG RNA production.
- We have reworded this section to be more precise: "In this assay, the nsP1-3 polyprotein and nsP4 are co-expressed with a reporter RNA that can be used as a template by the nsP4 RdRp, thus dissociating nsP4 enzymatic activity from nsP4 expression [53, 54]. Synthesis of the template RNA represents viral RNA synthesis and is detected by increased Firefly luciferase (Fluc) expression, while the RNA synthesized from the SG promoter is detected by *Gaussia* luciferase (Gluc) expression (Fig 3B)."

21. Fig 3: Panel A: add DAPI as separate panel, because it is present in the merged image. dsRNA is capitalized whereas nsP4 is not. Panel B: see Major Comment, does not correspond to Materials&Methods.
- We have added the Hoechst staining as a separate panel and corrected DsRNA to dsRNA. We have corrected panel B as described under major comments.

22. Line 218: add one sentence on how the ratio was determined using the ApaI restriction site.
- Done.

23. Lines 227-228: change the introduction (line 94) to include the contrasting results on the effect of C483Y on RdRp fidelity mentioned here.
- Thank you for the thoughtful suggestion. We have carefully considered this arrangement. However, we believe that including the contrasting results on the effect of C483Y on RdRp fidelity already in the introduction would be redundant. We feel it is more effective to present these contrasting results directly before our findings.

24. Line 236: the word frequency makes this sentence confusing, use 'number' instead.
- Done.

25. Fig 5: reduce the amount of white space. Panel B: the Y axis title seem wrong as it is the same as in panel A. Panel D: change legend to 1-5% and 5-10% instead of using the symbol '>'. Match colors to panels A-C.
- Done.

26. Line 259: add 'was determined by RT-qPCR'.
- Done.

27. Fig 6: To reduce white space, remove all CHIKV-labels in figure legend titles. Panels C, D: change X-axis title from 'Days post infection' to 'Days post inoculation' to match main text and other figures. Panel E: change u to µ. Change the 10 in log10 to subscript.

- We appreciate reviewer's suggestions. We have removed the CHIKV labels from this figure. We have updated the X-axis title to "Days post inoculation" as suggested. Regarding "log10," we believe it is already clear in its current format.

28. Line 285: add that this sentence refers to the reconstituted replication complex.
- Done.

29. Line 313: what is meant by 'isolation'? Please rephrase.
- Done.

30. Line 326-327: it is already clear that the finger domain is important for replication. Please, rephrase.
- Done.

31. Line 370: add antibody concentrations.
- Done.

32. Line 382: change 'prepared' to 'RNA was isolated'. Add whether the stocks were frozen in between the passages.
- Done.

33. Line 388: typo 'cycles'.
- Done.

34. Line 406: change 'in alignment with' to 'similar to'.
- Done.

35. Lines 422-428: which medium was used for the IF infections and were the cells washed after 1 hour?
- Done

36. Line 425: add antibody concentrations.
- Done

37. Lines 441-445: Were UMIs added to the library to increase the accuracy of the fidelity analysis and if not, why?
- UMIs were not incorporated into this specific fidelity analysis since there is no method to incorporate UMI-based analysis on a genome-wide basis. Instead, we used a genome-wide sequencing approach with high phred-score quality filtering, which enabled us to examine for reversion or compensatory mutations. We validated our approach to detect low frequency allelic changes on a molnupiravir treatment dataset previously generated in the lab (PMID 31645453).

REVIEWER 2

The manuscript by Yin et al describes the selection of 4'-FIU resistant CHIKV variants and their genetic and phenotypic characterization, both in cell culture and a mouse model. Mutations in the RdRp nsP4 were responsible for resistance against the chain terminator 4'-FIU. Mutations in nsP2 had a minor contribution. The paper convincingly demonstrates that the C483Y mutation (or other nsp4 mutation) does not affect fidelity, i.e mutation frequency in contrast to what the first study on this mutation claimed. However, later studies already suggested there was no strong effect of C483Y on fidelity. Therefore, the findings are not novel and the current study confirms the lack of a significant effect of C483Y and provides solid additional evidence. The antiviral effect of 4'-FIU was described before in an elaborate study by the same group. The current study does not provide much new information on the mode of action of 4-FIU or the mechanism involved in 4'-FIU resistance. The study is well performed and the results support the claims and conclusions of the manuscript. The properties, fitness and in particular the mutation frequency of the mutants is extensively characterized. The manuscript is concise and clearly written.

Major Comments

There were no comments listed here.

Minor Comments

1. The section of the results that starts at ln 177 is more discussion than results.

- We understand the reviewer's point. We presented some information on the nsP2 mutations here since we felt that it helped to explain why we do not further investigate them. The discussion instead focuses on the nsP4 mutations, including comparisons with prior findings, effects on fidelity, positions in the nsP4 structure, etc. We don't then circle back to the nsP2 mutations. To us, this is clearer, and we hope that the reviewer concurs.

2. The study is done with the attenuated vaccine strain 181/25 or the asian lineage strain AF15561, which is less pathogenic and exhibits decreased replication compared to other lineages like ECSA. Could the authors discuss this limitation. Could the use of these strains explain the lack of an effect on fidelity (for C483Y) in this study? As the first study that reported increased fidelity was done with an ECSA isolate. Authors could check the effect of C483Y in the ECSA background with their assays and compare it to the 181/25 results.

- The first statement about CHIKV strain AF15561 is not supported by data that we are aware of. Two other groups published studies suggesting that Asian strains are less pathogenic than an ECSA strain in mice (PMID 20519386, PMID 25995257). However, these studies were not performed with AF15561 and some of the differences reported were modest. Finally, we note that reference 56 also reported the lack of an effect on fidelity of the C483Y mutation in an ECSA strain (La Reunion).

3. Do the authors have an explanation why the resistance is not increasing over ~5-fold? Have they tried longer passaging and higher doses of compound? Did extra mutations emerge?

- We have not continued the selection for longer times or doses. The sequence analysis of P1-6 supports that the C483Y and Q192L mutations dominate during selection under our reported conditions. Based on the literature, nucleoside analogues do not tend to select for complete resistance, in keeping with our findings.

4. Fig 1D - Can it be concluded that the early Q192P mutant was outcompeted by the C483Y mutant? Is there experimental evidence for this, like for Q192L?

- We have not engineered this mutation into the infectious clone or performed direct competition experiments vs. C483Y. We have modified our comment in the discussion to make it clearer that we can only speculate on the competition of the other mutants vs. Q192L. "During the selection process, an nsP4 Q192P mutation arose first, but then appeared to be outcompeted by either the nsP4-C483Y or Q192L mutations."

5. Minor point: Fig 1C – nsP4-Q192L mutation listed in figure as Q912L

- Corrected.

REVIEWER 3

In this manuscript, Yin, et al establish that mutations in CHIKV that confer diminished sensitivity to 4′-fluorouridine (4′-FlU) at low to moderate concentrations do not confer a fitness advantage in vitro or in vivo, thus adding to the evidence that (4′-FlU) could potentially be used as a clinically effective antiviral treatment against CHIKV. To establish this, the authors first serially passage the attenuated CHIKV strain 181/25 and assess the mutations that arise over the course of passaging. They find and characterize multiple mutations that arise in the nsP2 and nsP4 replicase proteins in terms of fitness, dsRNA production, and 4′-FlU sensitivity in both mammalian and mosquito cell culture. In vivo data using the AF15561 strain clearly demonstrates that while two dominant

mutations lead to decreased sensitivity to 4′-FlU, these mutations do not confer a fitness advantage over wild type virus. This reviewer found the overall work to be of good quality, generally well written, and would be of interest to readers of Plos Pathogens. However, there are several points, particularly related to reporting of methods used, that if addressed would improve the manuscript.

Major Comments

1. The AF15561 strain was isolated some time ago. Are there AA differences in nsP2 or nsP4 across CHIKV strains- especially currently circulating or more recent clinical isolates? In figures 1D and 1F nsP2 mutations seem to occur concurrently or after nsP4 mutations. Could preexisting variances in more recent/clinically relevant strains already have some of these nsP2 mutations that might alter the interpretation of the data in this manuscript?
- The reviewer raises an interesting point. We now include alignments of nsP2/nsP4 across CHIKV strains, including contemporary strains, in the supplemental figures S2 and S4 to highlight the amino acid residues of interest. Note that residues nsP2-H687, nsP2-K704, nsP4-Q192, and nsP4-C483 are conserved across all of these CHIKV strains. We also now mention this conservation in the recent CHIKV isolates in the text.

2. Figure 1: Panel C was confusing due to the arrangement of the text above each Venn diagram. This should be adjusted so that the titles above each circle are easier to read and more closely associate with the circle they correspond to. The figure legend should also denote which colors represent the lineage mutations vs the plaque purified virus ones. The number of plaques that were purified from each lineage is missing. Please clearly state this.

- We have moved the titles above each circle so that they are more closely associated with the circle they correspond to. Additionally, we have updated the figure legend, indicated the colors, and included the number of plaques in both the main text and the figure legend.

3. Table S1: It would be helpful if there were a legend for this table. What does the "depth" column indicate? For lineage 2: the AA change is listed as C489Y whereas in line 137 it is denoted as C483Y. Is this correct or is the nucleotide change of nsp4: G1466A correct? In line 138, please clarify "with the total frequency of these two nsP4 mutations at ~90%". Do you mean that the sum of the frequency of having C483Y or Q192L was ~90%?

- For lineage 2, the AA change is C483Y, which has been corrected. We have also corrected the nucleotide changes of nsP4. We apologize for these typos. We have clarified the footnote defining the protein/position of the mutations. We have relabeled the "depth" column to "Sequencing Depth", and added a footnote to explain that this is the number of sequencing reads at that locus. We feel that with these changes an additional legend would be superfluous. Yes, we mean that the sum of the frequency of having C483Y or Q192L was ~90%. We have modified this in the text to "with the combined frequency of these two nsP4 mutations at ~90%".

4. Figure 2: Panel C and D- Line 172 states that a "low MOI" was used but the figure legend states an MOI=10. Please clarify. Please indicate what the early (pre-12hr) time points collected were. This is not stated or listed on graph.

- The cells were infected at a low MOI of 0.1 FFU/cell, which we have corrected in the figure legend. Adding the early time points (3 and 8 hpi) to the graph proved challenging due to limited space; however, we have listed all the time points in the figure legend.

5. Figures 3 and 4 were swapped. Figures will be referenced here as they were indicated in the text, not the uploaded figures.

- We have corrected, sorry for the inadvertent swap.

6. Figure 3: The schematic in panel B does not match what is stated in the methods (lines 430-432). Was a 2 or 3 plasmid system used for these experiments? Additionally, the timing indicated in the legend doesn't match what is stated in the methods.

- A 3 plasmid system was used for these experiments. We have updated the diagram in panel B and clarified the timing in the legend. See also reply to reviewer 1.

7. Figure 4:Ideally, it would have been nice to see at least one more biological replicate, but at minimum, please state what is plotted in these graphs. Are the % frequencies the averages of the technical replicates? The restriction digestion technique used for panels G and H needs substantial clarification. It was hard to make sense of how the data was generated and analyzed from the description in the text. A diagram and the inclusion of raw data, at least as an example in a supplemental figure, would be helpful in better evaluating these experiments.

- We appreciate the reviewer's comment. The % frequencies represent the averages of three technical replicates. We have clarified this in the figure legend. The method is described and referenced in detail in the methods section. To improve clarity we have added some details to the main text.

8. Figure 5: For panel A, please define what overall mutation frequency is and if there was, for example, a threshold set (ex 80% or higher) to determine what was counted as a mutation. It is difficult to understand how there could be more mutated sites than mutations. For panel C and line 237, please define Shannon entropy, how it was determined or calculated, and what it tells us. This was not specified anywhere in the text. The presentation of data in panel D was confusing and needs to either be shown in a different way or more thoroughly explained in the text. The figure key was also confusing. ">1%" of what? Should this also be >1 and <5, since all the >5 are also >1. Also, please move data reporting in legend (lines 868-869) to the results section. Please specify how statistics were done for panels E and F. Please define what * means in terms of significance in the legend.

- The overall mutation frequency was calculated as the number of mutated nucleotides per 10,000 mapped nucleotides after quality and depth filtering. There was no effective threshold set beyond the 300-fold depth at each reference base. We have added this definition "The overall mutation frequency was calculated as the number of mutated nucleotides per 10,000 mapped nucleotide after quality and depth filtering." to the legend for Figure 5A.
- Shannon entropy is a commonly used metric of diversity (or information). It was calculated using the diversity package in R off a matrix of the counts of A, C, T, G's at each locus across the CHIKV reference genome from deep sequencing data for each condition and replicate. If a polymerase mutation were more associated with error, it would give a higher Shannon entropy value. Our assessment was that there was no significant difference in this measure of mutation across the genome. We have added the definition of Shannon entropy and how it was calculated to the methods section.
- We have modified the figure key to 1-5% and 5-10%. To make the figure clearer we have matched the colors to panels A-C.
- We would prefer to keep this information in the figure legend, as it addresses the question raised by this panel.
- We have added the statistics description to panels E and F, and defined the *.

9. Figures S1 and S2: These figures need legends describing what the features are. What are the blue and black boxes? Please label or define in the legend what the numbers at the top are. What does TT stand for? Could the arrows and labeling of motifs be adjusted to be clearer which is which? It is hard to decipher. Also please indicate the strains of viruses from which the sequences were obtained.

- We have amended the figure legends to describe the features: "The Q192 and C483 residues are indicated by arrows, and the motifs in the nsP4 palm domain are marked by black boxes at the top of the sequences. The blue boxes showed the conserved regions. TT stands for tight turn." We have indicated the Swiss-Prot accession number of each virus in the figure legend.

Minor Comments

1. Line 196- "synthesis of the full length" was confusing. Both RNAs are going to be "full length".

- We have more fully described the assay and results in lines 224-230, and deleted the "full length".

2. Line 285- “the CHIKV replication complex does not contain viral RNA”- please make it more clear that this referencing the depicted structure and not the replication complex itself, which clearly contains viral RNA.

- We have modified it to “the reconstituted core structure of the CHIKV replication complex does not contain viral RNA”.

3. Line 295- while the motif D may not be conserved, it seems that C483 is completely conserved (Fig. S1). Please alter text accordingly.

- We have modified it to “while motif D is not conserved” and clarified that C483 is completely conserved.
